# Rock Microhabitats Provide Suitable Thermal Conditions for Overwintering Insects: A Case Study of the Spongy Moth (*Lymantria dispar* L.) Population in the Altai Mountains

**DOI:** 10.3390/insects13080712

**Published:** 2022-08-08

**Authors:** Grigory G. Ananko, Aleksei V. Kolosov, Vyacheslav V. Martemyanov

**Affiliations:** 1 FBRI State Research Center of Virology and Biotechnology VECTOR, Rospotrebnadzor, 630559 Koltsovo, Novosibirsk Region, Russia; 2 Laboratory of Ecological Physiology, Institute of Systematics and Ecology of Animals SB RAS, Frunze str. 11, 630091 Novosibirsk, Russia; 3 Biological Institute, National Research Tomsk State University, 634050 Tomsk, Russia

**Keywords:** winter survival, overwintering microhabitat, rock microclimate, extreme temperatures, supercooling points (SCP), oviposition, gypsy moth, spongy moth, alpine environments

## Abstract

**Simple Summary:**

The spongy moth (SM) lives in the temperate latitudes of Europe and Asia, as well as in North America (as an invasive species). Caterpillars of the spongy moth feed on tree leaves. They cause significant economic damage, as they lead to the defoliation of trees over vast areas. Studies of the ecology of the spongy moth are necessary to predict possible outbreaks of this insect, as well as to develop effective methods for controlling their numbers. In North America, where the SM is an invasive species, its range is still expanding. Therefore, it is vitally important to build adequate models that will help predict the regions where the SM may appear in the future. The results of our study of Asian SM populations suggest another possible pathway for the SM to spread in the forests of North America. It turns out that the temperature of the rock surface, where the spongy moth lays eggs overwinter, is significantly higher than the temperature of the winter air. Therefore, insects (or their eggs) wintering on rocks can survive colder winter air temperatures than their physiology allows. These results help to reassess the role of the mountain landscape in the spread of insect species.

**Abstract:**

Many insect species overwinter in various rock shelters (cavities and crevices), but the microclimates of rock biotopes remain poorly understood. We investigated the temperature dynamics in rock microhabitats where clusters of egg masses of the wintering spongy moth *Lymantria dispar* L. (SM) were observed. Our research objective was to find the relation between the ovipositing behaviour of females and the landscape features in different parts of this species’ range. Studies of the ecology of the SM are important from a practical point of view, as the moth causes significant economic damage to forests of the Holarctic. We found that the average monthly temperature of rock surfaces in the studied microhabitats was 2–5 °C above the average air temperature. More importantly, the minimum temperatures in these microhabitats were 4–13 °C higher than the minimum air temperature. These results help to reassess the role of the mountain landscape in the spread of insect species. Rock biotopes provided a significant improvement in the conditions for wintering insects. We believe that, when modelling the spread of invasive species (such as the SM), it is necessary to account for the influence of rock biotopes that may facilitate shifts in the northern boundaries of their range.

## 1. Introduction

Insect species inhabiting temperate and polar latitudes survive the winter in a dormant state [1,2,3]. They use various physiological and behavioural adaptations that allow them to survive adverse environmental conditions such as low temperature and humidity [4,5]. Known physiological mechanisms of cold resistance include the synthesis of cryoprotectants (polyatomic alcohols and sugars), antifreeze proteins and dehydration [1,6,7]. In the strategy of wintering insects in harsh continental climates, the choice of microhabitats is no less important than the physiological mechanisms of survival [2,8,9,10]. Insects often overwinter under stones or in the cracks and crevices of rock outcrops. However, there are few studies of the temperature conditions of insect wintering sites in rocky areas [11,12,13]. The lack of accurate ecological data on local microhabitats is one of the factors limiting the integration of physiological and ecological information [14].

Resistance to low temperatures is a key component of the fitness of poikilothermic animals and one of the most important factors determining their distribution [15]. Knowledge of the mechanisms that determine the temperature in microhabitats preferred by wintering insects is important for solving a wide range of theoretical and practical problems. These include preventing vector-borne human diseases through controlling the spread of insect vectors, forecasting the dynamics of pest insect outbreaks in forestry and agriculture, and forecasting possible changes in the boundaries of insect ranges associated with climate change and the spread of invasive species. Forecasting the spread of the spongy moth *Lymantria dispar* (SM) in North America is complicated by the fact that the SM is an invasive species and the size of its range in the forests of the USA and Canada continues to increase [16,17].

The European subspecies is naturalised in Eastern North America and causes severe outbreaks whereas the Asian subspecies is not yet established, and effort are on-going to keep it out of the continent. The spongy moth *Lymantria dispar*, which is one of the most significant pests in Holarctic forests, was the object of our research. The spongy moth (synonym—gypsy moth) is included in the list of the 100 worst invasive alien species in the world. An urgent task in applied ecology is to predict outbreaks in the numbers of this insect and possible changes in the boundaries of its range associated with climate change [16]. Due to the plasticity in the parental behaviour of females, the Asian subspecies, *L. dispar asiatica,* is a suitable case study for examining the role of behavioural adaptations in insect ecology [18]. The vast range of this insect is characterised by a wide variety of climate, terrain and vegetation factors. The behavioural strategies of different populations of SM also differ correspondingly. Females of the European subspecies of SM (*L. dispar dispar*), which are also common in North America, do not fly, whereas females of the Asian subspecies of SM (*L. dispar asiatica*) are capable of flight [19]. Within the Asian subspecies, some scientists distinguish four geographical forms that differ in food preferences and parental behaviours of females during oviposition [20]. Many studies have been devoted to the ecology of the SM, however, it remains unclear whether the parental behaviour of the female is an optimal strategy when choosing microhabitats for oviposition.

The spongy moth belongs to the group of univoltine insect species that hibernate in the egg stage. The survival of the SM depends to a large extent on the conditions of the previous winter and the resistance of SM eggs to negative temperatures [21]. The eggs of the spongy moth use a cold-resistance mechanism known as freeze avoidance [22]. Freeze-avoiding insects undergo a series of physiological changes, including removal of all potential nucleating agents, synthesis of polyols, sugars and antifreeze proteins, allowing to stabilise the supercooled state [6]. The preservation of the supercooled state of insect eggs is facilitated by their small size, the absence of crystallization centres [6,7], as well as the presence of the cryoprotectant, glycerine, in SM eggs [22]. It is known that the lower limit of the cold hardiness of the eggs of *L. dispar dispar* corresponds to approximately −30 °C [21,23,24,25,26]. The eggs of *L. dispar asiatica* have been long believed to be more resistant to cooling than *L. dispar dispar*, as some have survived cooling to −48 °C [27]. However, a recent study [28] showed that the cold hardiness of Asian populations is close to that of European populations: the lower limit of cold resistance of the three studied Asian populations (Novosibirsk, Altai, Kyrgyz) ranges from −29 °C to −30 °C. Moreover, the supercooling point is sometimes suggested as a factor in the prediction of range expansion of this species [26], while the actual survival of this species could be significantly modified by its behaviour.

In the SM range in the Asian part of Russia (Siberia, a substantial part of the Far East) during a typical winter, the temperature repeatedly drops below −30 °C (sometimes below −40 °C) (www.pogodaiklimat.ru, accessed on 1 January 2021). Consequently, the temperature tolerance limits of SM eggs are insufficient for survival in such extreme conditions. The lack of physiological stability is assumed to be compensated by the flexibility of parental behaviour of females of the Siberian populations [20]. Thus, females of the Western Siberian populations of SM, as a rule, lay eggs at the bases of tree trunks, with an average oviposition height of 6–7 cm from the soil level [20,29]. This behaviour is explained by the presence of stable snow cover from November to March in Western Siberia. Snow cover is a good thermal insulator: temperature fluctuations are much less pronounced under snow in comparison with the air temperature above the snow cover [21,24]. Thus, the presence of a stable snow cover makes it possible for SM eggs to avoid exposure to the atmosphere, the temperature of which is below the supercooling point of diapausing SM eggs, in extremely cold Siberian winters.

However, SM populations also successfully winter in several mountainous regions in Eastern and Southern Siberia, where the weather is variable and in most years there is less snow than in flat areas. Ust’-Kan District of the Altai mountains, where we conducted studies of rock biotopes, is such a region (www.pogodaiklimat.ru, accessed on 1 January 2021). A distinctive feature of the mountain populations of the SM is the specific choice of biotopes for oviposition: most females prefer to lay eggs in the cavities and crevices of rock outcrops, even if there are suitable trees nearby [19,20,29,30,31]. The reasons for this behaviour remain unknown. One of the possible explanations is that laying egg masses on rocks (exposed to wind) increases the spread of emerging young caterpillars [32], which could be important for SM genotype exchange [33]. The fact is that the hatched larvae release cobweb threads and are subsequently carried by the wind [17]. According to another hypothesis, the preference for the southern slopes of rocks accelerates the hatching of caterpillars, since the southern slopes are better warmed by the sun [13,20]. These possible advantages have not been experimentally verified.

We proposed that rock biotopes possess one more advantage that is critically important for the survival of SM eggs under continental winter conditions: The possible difference in temperature between the rocks and the surrounding air could explain the survival of eggs in cold winter conditions. The purpose of this work was to study the rock biotopes used by insects for wintering and to clarify the limits of resistance of *L. dispar asiatica* eggs to low temperatures using an integrated approach. The spectrum of supercooling points of SM eggs collected at the observation sites was studied under laboratory conditions. In the field, the temperatures of the main rock biotopes, where SM eggs were oviposited, and the ambient air temperatures were constantly recorded. In particular, we tried to answer the following questions: What are the limits of the physiological resistance of the eggs of the Altai SM population to low temperatures? Does the local temperature of the rock surface, in places where eggs of the Altai population of the SM are accumulated, differ from the ambient air temperature? Does wintering on rock outcrops contribute to egg survival?

## 2. Materials and Methods

### 2.1. Field Studies

Field observations were conducted in the Ust’-Kan District of the Altai mountains (Russia) in the valley of the Anuy river, near the village of Cherny Anuy (51.33° N, 84.74° E, 808 m above sea level). The nearest meteorological station is located in the village of Ust’-Kan (50.93° N, 84.75° E, 1037 m above sea level), 90 km from the study site. Therefore, we recorded the air temperature (in the shade) next to the rock microhabitats.

Egg masses from the Altai population of the SM were collected twice: On 24 September 2019, to test the initial viability of eggs and determine the supercooling point (SCP), and on 24 April 2020, to test the viability of eggs after wintering in rock microhabitats. According to Ponomarev et al., 2012 [20], the Altai population belongs to the East Siberian geographic form. The range of the East Siberian geographic form is extensive and covers deciduous and larch forests from Altai in the west to the southern foothills of the Stanovoy Range in the east, Mongolia and Khingan.

**Figure 1:**
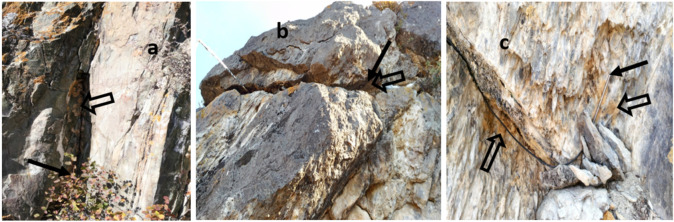
Sites where temperature sensors were installed (51.33° N, 84.74° E, 808 m above sea level): (**a**)—a recess in the rock (“recess”), (**b**)—a slit under the stone at a depth of 20 cm from the edge (“slit”), (**c**)—a flat rock surface (“flat surface”). The solid black arrows indicate the locations of the sensors, and the outlined, transparent arrows indicate the SM egg masses on the rock.

Loggers with temperature sensors were installed on the rocks where multiple SM egg masses were found. Almost all the egg masses we found were located on the southern side of the rocks, while only a few egg masses were found on slopes with western and eastern exposures. The temperature was recorded at 4 points. The first temperature sensor was located in the narrowest part of the recess of the rock (Figure 1a) so that the ledge of the rock on its left shaded it from direct sunlight (hereinafter referred to as the “recess”). The second sensor was located in a narrow slit underneath a large stone (Figure 1b) at a depth of 20 cm from the edge of the slit (hereinafter referred to as the “slit”); this microhabitat had more egg masses than other microhabitats. The third sensor was located in the shadow of this stone (on the north side), measuring the temperature of the air near the rock (10 cm from the surface of the rock). The last sensor (Figure 1c) was located on the open surface of a flat rock exposed to the sun (hereinafter—“flat surface”); individual egg masses were also found here, but there were significantly fewer of them than were observed in various recesses and cracks. The same photo (Figure 1c, arrow on the left) shows that many more egg masses were located under overhanging ledges of rocks. The fourth sensor (“air—near-rock layer“) recorded the air temperature (in the shade): located behind a stone (Figure 1b), a few meters from the sensors that record the temperature of the rock surface. To record the temperatures, Relsib 2-channel EClerk-M-11-2pt-HP loggers were used (https://relsib.com/product/izmeritel-registrator-temperatury-eclerk-m-2pt-hp, accessed on 1 January 2019), the temperature determination error of which was is 0.2 °C. Temperature sensor readings were recorded every hour.

### 2.2. Determination of the Viability of SM Eggs

Prior to testing for viability and cold resistance (in December 2019), egg samples collected in the fall were stored in the refrigerator at 6 °C. Immediately prior to the experiments, the eggs were cleaned of their hair coating and placed in a plastic Petri dish with a diameter of 10 cm. Eggs were incubated at 26 °C, with a relative humidity of 60% and a 16 h day/8 h night regime. Hatched caterpillars were counted daily. We counted the hatched larvae every day and continued the experiment for one week after all larvae had hatched, but not less than 4 weeks. Experiments were repeated in 5 replicates (with 100 eggs in each) for each microhabitat.

### 2.3. Determination of the Supercooling Point (SCP)

For the experiment, a random sample of eggs from several hundred egg masses of the Altai population of *L. dispar asiatica* collected in several dozen rock microhabitats was used. When collecting egg masses, we sought to ensure maximum genetic diversity in the experimental egg samples, so no more than 5 egg masses were taken from one site. Before starting a series of experiments, the eggs were cleaned of their hair coating and thoroughly mixed to obtain a random sample of eggs.

The egg masses were stored at a temperature of 6 °C. Before each series of experiments, the SM eggs were cleaned of their hair coating. These experiments were conducted in December, since by this time the eggs must have reached the maximum cold resistance in order to survive the winter. The two installed PT1000 sensors had an accuracy of ±0.05 °C and a measurement range between −50 and +100 °C. Batches of eggs (*n* = 20 eggs/batch) were attached to the surface of the main temperature sensor (PT1000) with adhesive tape. The main sensor (with attached eggs) and the second sensor (reference) were placed in polypropylene tubes (1.5 mL volume). The test tubes themselves were immersed in a vessel with 60% glycerine. In turn, the vessel with glycerine was placed in a special thermal insulation chamber in a low-temperature freezer (set at −35 °C) to slowly cool the eggs at a rate of 1–2 degrees per hour. The thermal insulation chamber had hollow walls, inside of which a 60% glycerine solution was poured, and, in addition, the inside of the chamber was thermally insulated with foam and cotton. The sample chamber was cooled in a low-temperature freezer, and data on the temperature of the samples was transmitted via cable to a recorder placed next to the freezer every 2 s. Sudden temperature peaks on the main sensor indicated the release of energy during the crystallization of water in the eggs [6].

### 2.4. Rock Microhabitat Simulation Experiment

The experiment was conducted from 11–16 February 2021, in the suburbs of Novosibirsk. One sensor was placed directly on the outer surface of a concrete wall of a house, and the second sensor was located 3 cm from the wall (and from the first sensor) to measure the temperature of the near-wall air layer. The wall faced south, but the sensors themselves were in the shadow cast by the balcony railing. To record the temperatures, a Relsib 2-channel EClerk-M-11-2pt-HP logger was used. Temperature sensor readings were recorded every 10 min.

### 2.5. Statistical Analysis of the Results

During field studies, temperature values in microhabitats were recorded every hour. Data on the minimum daily temperatures during the observation period are shown on the graph. The monthly temperatures for October–November 2019 are given in table form: the mean temperature, average monthly minimum temperatures, overall maximum and minimum temperatures, fluctuation amplitude of the monthly temperature. The significance of differences in samples of average daily temperatures (“rock microhabitats” vs. “air”) were evaluated using a nonparametric chi-squared criterion. The Mann–Whitney U test was used to access the significance of differences between the minimum daily air and rock temperatures: the observational data for November–December 2019 were divided into groups with an interval of 3 days (*n* = 19). To test the hypothesis of the connection of air temperature with the temperature difference, we performed separate linear regressions temperature difference (between the rock/wall and the air) against air temperature. To assess the viability of the SM eggs, 5 egg masses were selected from each microhabitat and 100 eggs from each egg mass were left to hatch. Two parameters were evaluated for the eggs from each microhabitat: the average viability and the timing of the beginning of hatching. The significance of the differences in the average viability and hatching time of eggs from different microhabitats was assessed using a *t*-test. To account for multiple comparisons in these tests, the Bonferroni correction was applied. The median supercooling point was calculated based on the supercooling points of 100 eggs of the Altai population. Quantitative data were presented as arithmetic means ± standard error of the means. The STATISTICA Advanced software package was used to process the results (http://statsoft.ru/products/STATISTICA_Advanced/, accessed on 1 January 2022).

## 3. Results

### 3.1. Distribution Spectrum of Eggs of the Altai Population by SCP

**Figure 2:**
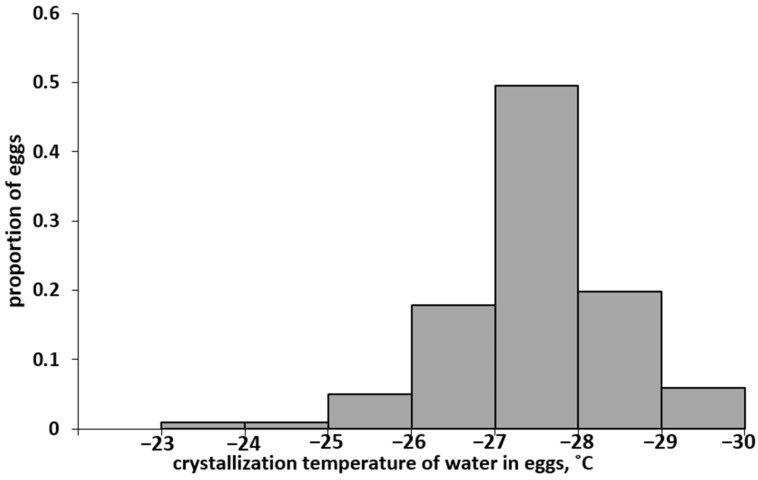
Distribution spectrum of eggs of the Altai SM population according to the supercooling points (SCP).

In a series of experiments, the values of supercooling points were obtained for 100 eggs of the Altai population. The SCP values varied in a wide range, from −23.5 to −29.4 °C. The distribution spectrum of eggs of the Altai population of *L. dispar* is shown in Figure 2.

The arithmetic mean SCP was −(27.5 ± 0.1) °C and coincided with the median, i.e., half of the eggs in this population died when cooled to −27.5 °C. Interestingly, this value almost exactly coincided with the previously established [28] LT_50_ value of −27.4 ± 0.2 °C; even with short-term cooling to the specified temperature, 50% of the eggs of the Altai population died. The coincidence of the SCP_50_ and LT_50_ values suggests that the eggs of the Altai population died as a result of damage caused by the formation of crystals.

### 3.2. Characteristics of the Climate in the Observation Area

**Figure 3:**
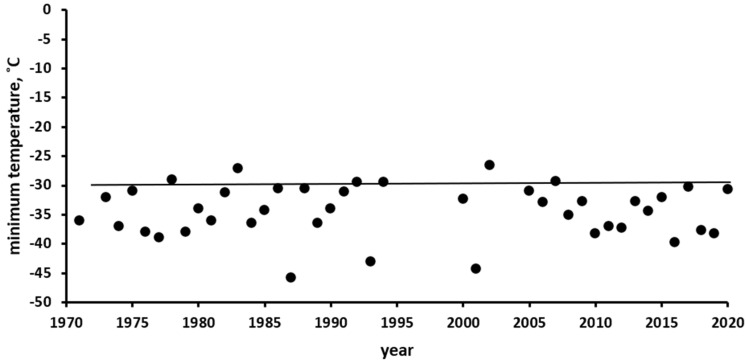
Minimum air temperatures by year, according to the weather station in Ust’-Kan (50.93° N, 84.75° E, 1037 m above sea level) (http://www.pogodaiklimat.ru/history/36213.htm, accessed on 1 January 2021). The solid line corresponds to the critical negative temperature that is lethal to the eggs of the Altai SM population.

The Ust’-Kan District of the Altai Mountains, where the observations were conducted, belongs to a region of Russia with a harsh continental climate, where winter temperatures reach extremely low values. In Figure 3, compiled according to the data of the Ust’-Kan weather station (http://www.pogodaiklimat.ru/history/36213.htm, accessed on 1 January 2021), it can be seen that the minimum annual temperatures were higher than −30 °C only twice in 50 years. During winter, the air temperature usually repeatedly falls below this mark, and sometimes it even falls below −40 °C.

Little snow falls in this area during the winter months (December–February), with 5–7 mm of precipitation per month on average since 1970 (http://www.pogodaiklimat.ru/history/36213_2.htm, accessed on 1 January 2021). In addition, precipitation is unevenly distributed over time, so, during our observations (October–December 2019), there was no snow cover, and a small amount of snow fell only in January 2020.

### 3.3. Comparison of Air Temperature and Temperature in Rock Microhabitats

We found out that in winter (November–December), the surface temperature of the rocks is significantly (χ^2^ = 215, df = 7, *p* = 6.34 × 10^−43^) higher than the air temperature in the shade. The greatest differences were observed for the “slit” and “recess” microhabitats. However, even on an open flat rock surface (“flat surface”) the temperature was higher than the air temperature (χ^2^ = 84, df = 7, *p* = 8.17 × 10^−16^). During the observation period, from October to December 2019, the average monthly temperatures in the rock biotopes were 2–5 degrees higher than the average monthly air temperatures recorded in the near-rock layer of air (in the shade) (Table 1).

**Figure 4:**
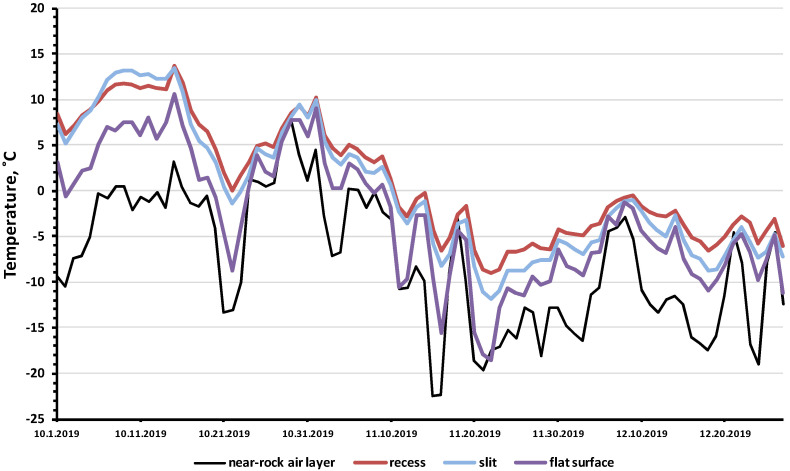
Minimum daily temperatures in three rock microhabitats in comparison with the air temperature near the rock layer in the shade (October–December 2019).

A particularly large difference was observed in the minimum temperatures: in the three studied rock microhabitats, the minimum temperatures were 4–13 degrees higher than the recorded minimum air temperatures in the near-rock layer of air (Figure 4, Table S1). The analysis of samples of the minimum daily air and rock temperatures (Table 2) showed that the temperature differences between the rock biotopes and the air temperature were highly significant (U = 46, *p* = 0.00036). For the entire observation period, the minimum temperature of the rock microhabitats was −18.5 °C, while the minimum air temperature was −22.5 °C.

**Figure 5:**
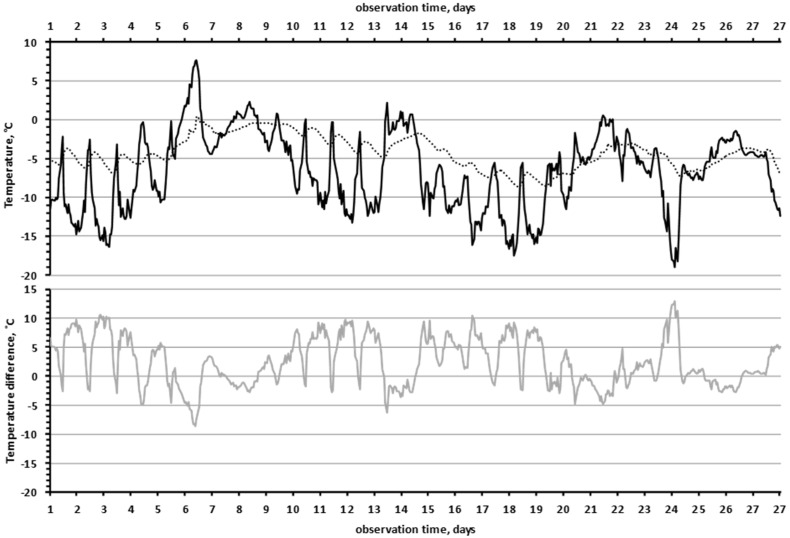
The lower the air temperature, the greater the temperature difference between the rock and the air (December 2019). The temperature of the air in the shade, 10 cm from the surface of the rock (black line). The temperature in the “slit” (dotted line). The temperature difference between the rock in the “slit” and the air (grey line).

Another interesting effect was that the lower the air temperature was, the greater the temperature difference between “the near-rock air layer” and the “slit” in the rock. In Figure 5, we see that the black curve (“near-rock layer air temperature”) was mirrored by the grey curve (temperature difference between “near-rock layer air temperature” and the “slit”). This effect was highly significant, since the correlation coefficient between the values of air temperature and the temperature differences (between the “slit” and the air) was −0.91 (df = 647, F = 3059, *p* = 2 × 10^−247^).

A comparison of the amplitude of temperature fluctuations showed that, in December, in the “recess” and “slit” microhabitats (Table 1), the difference between the maximum and minimum temperature was relatively small (9–11 degrees), while the amplitude of air temperature fluctuations was 26–29 degrees. Thus, rock microhabitats mitigated temperature fluctuations: the amplitude of the temperature fluctuations of the rocks was 2.6–3.2 times less than the amplitude of the air temperature fluctuations. It should be noted that, on the open surfaces of flat rocks (“flat surface”), the amplitude of temperature fluctuations was almost as high (22 degrees) as that of air. On the one hand, the rock was heated by solar radiation, increasing the temperature of the rock on sunny days, and on the other hand, the flat surface of the rock experienced more intensive cooling by cold air flows.

### 3.4. Viability of Overwintered SM Eggs

The samples of egg masses collected in April 2020 were left to hatch immediately after collection (Table 3). Caterpillars from the “flat surface” microhabitat began hatching earlier than caterpillars from other microhabitats; some hatched within one day of collection. This was presumably because this surface was directly warmed by the sun, unlike other microhabitats in the shade. Nevertheless, a day later, the first caterpillars also hatched in the samples from the “slit” and “recess” microhabitats. Thus, the proportion of viable eggs did not differ significantly between habitats.

In addition to samples from places where temperature sensors were installed, data on the hatching of caterpillars from samples collected from an additional “deep recess” site (55 cm) on the eastern slope were also of interest (Table 3, last column). Several egg masses were found in this microhabitat. The viability of the eastern slope eggs did not differ significantly from viability of the southern slope eggs, but the time before hatching was significantly higher in the eggs of the eastern slope. The caterpillars in the eastern microhabitat began to hatch only 91 h after collection, i.e., hatching started 63–77 h later than in the southern microhabitats. These results support the hypothesis that the southern exposure of the egg masses allowed the caterpillars to hatch much earlier.

### 3.5. Rock Microhabitat Simulation Experiment

**Figure 6:**
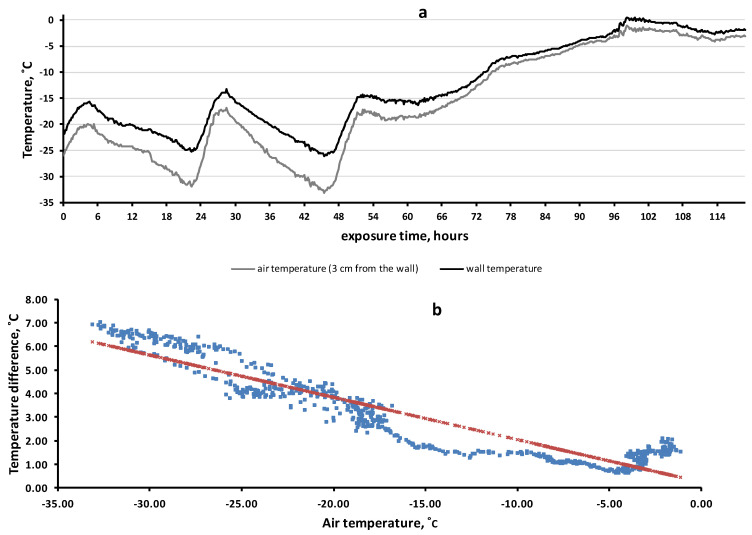
Temperature dynamics of the wall surface of the house and in the near-wall air layer (11–16 February 2021) (**a**). The regression of temperature difference (between the wall and the air) by air temperature (**b**).

To clarify the factors determining the temperature of the rock surface, we set up a simulation experiment. The results are shown in Figure 6. The wall of the house quite accurately imitated rock surfaces. First, the coefficient of thermal conductivity of reinforced concrete (1.7 B_T·M_^−1^·K^−1^) approximately corresponded to that of rocks, 1.1–3.9 B_T·M_^−1^·K^−1^. Second, there was a heat flow from the inside of the wall to the outside due to the temperature difference inside and outside the room. During the observation period, the average wall temperature (−12.8 °C) was 3.1 degrees higher than the average air temperature (−15.9 °C) (χ^2^ = 193.6, df = 8, *p* = 6.83 × 10^−38^).

The adequacy of the model experiment was also evidenced by its results: the difference in average monthly temperatures between the flat surface of the rock (“flat surface”) and the near-rock air layer (Table 1) was also 1.6–3.1.

Figure 6 allows us to demonstrate another effect that we have observed in rock biotopes: the lower the air temperature was, the greater the difference between the temperatures of the wall and the air. This effect was highly significant: R = −0.937 (df = 712, F = 5143, *p* = 4 × 10^−306^). Thus, at 45 h of exposure time, the air temperature was −33.1 °C while the wall temperature (−26.2 °C) was 6.9 °C higher. However, during the thaw period (90–120 h), when the air temperature ranged from −5 C to 0 °C, the average temperature difference between the wall and the air was only 1.4 °C (varied from 0.7 to 2.1 °C).

## 4. Discussion

### 4.1. Climate and Microclimate in the Research Area

Understanding how insect species adapt to extreme winter temperatures is an important step towards predicting their response to climate change. However, the actual microclimate in local biotopes may have significant differences from a region’s macroclimate. The temperature in a particular microhabitat depends on a complex combination of local factors, and in particular, it may differ from the air temperature due to the presence of snow cover and the influence of soil [34]. Therefore, the temperature values recorded at meteorological stations where Stevenson screens are used do not always adequately reflect the temperature conditions in microhabitats where insects overwinter [2,11].

The range of *L. dispar* is very large in area (significant part of the Holarctic region) and, accordingly, it is characterised by a wide variety of landscapes and climatic conditions. At the same time, different populations have a similar resistance to extreme low temperatures. Thus, most researchers on the European subspecies *L. dispar dispar* (also introduced to North America) agree that the lower limit of the temperature stability of the SM eggs is approximately −30 °C [21,23,24,25,26]. According to the latest data [28], the lower limit of the temperature resistance of Asian populations of SM (*L. dispar asiatica*) is close to that of the European subspecies, also approximately −30 °C. The results of the study on the spectrum of SCP values of the Altai population described above (see Figure 2) confirm this conclusion using an independent method. Stable populations of SM exist in Siberia, although the winter temperature in this region repeatedly drops below −30 °C and sometimes below −40 °C. In particular, in the Altai Mountains where we conducted observations, the minimum winter air temperatures (Figure 3) fall below the temperature stability limit of the SM eggs every winter, with a few exceptions. Thus, there is an obvious discrepancy between the physiological resistance of *L. dispar asiatica* eggs to negative temperatures and the climate of the region.

### 4.2. The Relationship of Parental Behaviour of the SM with the Climate and Landscape of the Region

For those insect species, such *L. dispar asiatica*, whose physiological stability limits are narrower than the range of winter air temperature fluctuations, an adequate choice of microhabitat is critically important for survival [2,8,9,10]. The diversity of ecological preferences shown by different populations of SM [19,20] illustrates this thesis well. The results of our study showed that the SM eggs deposited on the surface of rocks survived due to the specific microclimate in these biotopes. On the surface of the rocks, the average monthly temperatures (in different microhabitats) were 2–5 °C above the average winter air temperature. More importantly, the minimum temperatures in the rock biotopes were 4–13 °C higher than the minimum air temperature. Rock outcrops have powerful buffer properties to maintain temperature, which allow daily and seasonal temperature fluctuations to be neutralised. Buffer properties are especially pronounced in areas with harsh continental climates, allowing the temperature regime of the rock biotopes to be maintained within the physiological limits of the insect. This is confirmed by our data on the survival of wintering SM eggs. Our data also indicated the suitability of the parental behaviour of Altai SM females. At extreme winter air temperatures (which are lethal to the SM eggs), and in the absence of snow cover, the surface of rocky outcrops provided acceptable conditions for wintering eggs.

In addition to the East Siberian geographical form we studied, a number of researchers [20] distinguished three more geographical forms of *L. dispar asiatica*: West Siberian, Far Eastern and Central Asian. These groups use different behavioural strategies when laying eggs to go through the winter diapause. Thus, egg masses of the West Siberian geographical form are located at the base of trunks, close to the soil [20,29,30]. This behaviour is appropriate in the western Siberian climate, where a stable snow cover is usually established from November to March. Snow acts as a heat insulator, protecting eggs from extreme winter temperatures [25,35]. For example, in Japanese populations of the spongy moth, the height of the snow cover was one of the limiting factors in the ecological success of the insect [33]. In areas where snow cover is insignificant or unstable, the spongy moth uses alternative behavioural strategies. Females of the Far Eastern geographical form oviposit on the underside of the leaves. After the leaves fall in autumn, the SM egg masses end up in the litter on the soil surface: plant debris is thought to protect eggs from extreme winter temperatures, even in conditions of unstable snow cover [36,37].

In addition to insects, many other species of animals and plants live in rock biotopes. In one of the few studies of mountain biotopes so far, Conver et al. [38] recorded the air temperature in the stone cavities where saguaro cactus (*Carnegiea gigantea*) grew. They found that the rocks mitigated the effects of negative temperatures: the winter air temperature in the crevices of the rocks where the cactus grew was on average 2 degrees higher than in the open control areas. In addition, they noticed that the protective effect of rocks increased as the air temperature decreased. However, Conver et al. [38] did not study the temperature of the rock surface. According to our data, in December, the average temperature of rocks in different biotopes was 2–3 degrees higher than the temperature in the near-rock air layer (Table 1). The difference between the minimum temperatures of air and rocks (5–10 degrees) was even greater.

There are trade-offs in choosing a suitable place for wintering, with many favourable and unfavourable consequences of each choice. On the one hand, shelters are useful, since in a hibernation state, insects become inactive and immobile, and they cannot actively avoid predators, parasites and unfavourable climatic conditions. Many parasites use chemical signals from plants (volatile compounds) for primary navigation when searching for their host. For example, there is a spatial gap in the interaction between the offspring of silkworms wintering on rocks and their parasites [39]. On the other hand, in open areas, insects emerge from diapause faster in the spring, as they are exposed to more solar radiation [20]. Thus, our study revealed a significant difference in the hatch dates of caterpillars in microhabitats with eastern and southern exposures.

### 4.3. On the Mechanisms of “Warming” of Rock Microhabitats

**Figure 7:**
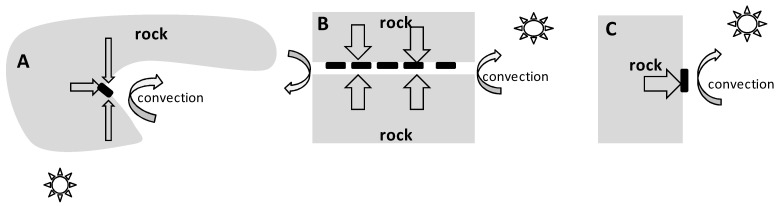
Cross-sectional diagrams of microhabitats. (**A**) is a recess in the rock, triangular in section (“recess”); (**B**) is a slit through the rock (“slit”); (**C**) is a flat rock surface (“flat surface”). The black rectangles indicate the locations of egg masses on the surface of the rock. The straight arrows indicate the heat flow vectors from the inside of the rock to its surface. The curved arrows indicate the influence of air flows. The sun shows the source of solar radiation.

The surface of the rock is a unique microbiotope located on the border of rock and air, which sharply differ in properties. In temperate and polar latitudes in winter, the average temperature inside rocks is higher than the average air temperature [40]. Therefore, the heat flow vector is directed from the inside of the rock to its surface, i.e., in the direction of decreasing temperature (Figure 7).

An object located on the surface of the rock (e.g., egg mass, temperature sensor) is heated by the rock on one side and cooled by moving streams of cold air (convection) on the other side. In this case, the temperature of the object results from the balance between two heat flows and has an intermediate value between the temperature of the air and the rock. The thermal conductivity of the main rocks composing the Anui Ridge (limestone, shale and granite) varies from 1.1–3.9 B_T·M_^−1^·K^−1^ [41], and the thermal conductivity of the air is ~0.02 B_T·M_^−1^·K^−1^ [42], i.e., two orders of magnitude lower. Thus, heat is transferred more efficiently from the rock to the object on the surface than from the object to the air. As a result, the average and minimum temperatures of objects in close contact with rock surfaces are higher than those of the air.

The higher heat capacity of rocks allows them to act as accumulators, absorbing thermal energy during the day and releasing it at night [43]. On a yearly scale, a similar process takes place: during the summer season, rocks accumulate energy, and in winter, they release it. The air temperature can change quickly, whereas the rock reacts very slowly to changes in air temperature, thanks to its very large mass. As a result, we see (Figure 5) that the lower the air temperature is, the greater the temperature difference between the air and the slit in the rock. Thus, the buffer effect of rock biotopes is more pronounced in regions with cold winters, neutralizing the harmful effects of the extreme decrease in air temperature.

The temperature of objects adjacent to the rock surface is due to the interaction between a number of factors: heat flow from inside the rock to its surface, cooling of the rock by air flows, and direct heating of the rock by solar radiation (Figure 7). In the recesses and slits, as observations have shown, the temperature of the rock surface is even more different from the air temperature. Most likely because the air is less mobile in the slit (Figure 7B), the cooling due to convection is not as intense as it is on open surfaces. In addition, the air in the slit is simultaneously heated by the rock from above and below. Another probable reason is that objects located in the slits (Figure 7B) and recesses (Figure 7A) are closer to the inner layers of the rock, where the temperature is higher.

Interestingly, in the summer, rock biotopes also exhibit buffering properties, but are used by poikilothermic animals for cooling. The reason is that in summer the temperature inside the rock is lower than the air temperature: the animal’s body, closely adjacent to the rock, cools faster due to the higher (than air) thermal conductivity of the rock. It was shown that spiders [44,45] and snakes [46] choose stones of a suitable size for shelters to maintain optimal body temperature.

The buffer properties of rocks are similar to the influence of such a factor as soil. The soil protects animals wintering within it from the effects of extremely low air temperatures [47,48,49]. In winter, the heat flow vector is usually directed to the soil surface and, as a result, the temperature of the upper soil layer is significantly higher than the temperature of the winter air. However, the thermal conductivity of the soil varies very widely, as it depends on its mineralogical and granulometric composition, air content, humidity, density and content of organic residues [50,51]. In contrast, the thermal conductivity of rocks is more stable, providing a more predictable microclimate for insects wintering on their surface.

The results of the simulation experiment (see Figure 6) indicate that laying eggs on the walls of houses is also an effective strategy, since the average and minimum temperatures of the walls are significantly higher than the air temperature in winter. Various researchers have repeatedly observed insect eggs on house walls, fences and poles in places of mass SM outbreaks [36,37].

### 4.4. Advantages of Rock Microhabitats

According to our observations, females of the Altai population prefer to lay eggs on rocky outcrops. This is also observed by Benkevich [30] and Hauck et al. in Mongolia [31], on the eastern border of the range of the East Siberian geographical form of *L. dispar asiatica*. The greatest concentration of egg masses is observed, not on open surfaces, but in various cracks or crevices. At the same time, clusters of egg masses are in the shade most of the day, without direct exposure to solar radiation (Figure 1), which can be a limiting factor in the summer months when the temperature on the surface of the rocks can be lethal.

Laying egg masses on rock outcrops has been hypothesised to increase the spread of newly hatched caterpillars in spring, since wind blows freely against the rocks [32]. The hatched larvae release cobweb threads and are subsequently carried by the wind. This is the main method by which the SM spreads, but there is no consensus on how far the wind can carry the caterpillars [17,33,52].

Another hypothesis focuses on the fact that the slopes of mountains with southern exposures warm up faster in spring, accelerating the hatching of caterpillars. This, in turn, may achieve a maximum synchronization of the growth rate of the population’s caterpillars with the phenology of their forage plants [13,20]. The hypothesis that caterpillars from egg masses located on the southern slopes hatch earlier was confirmed by the results of our study. We found out that eggs collected from microhabitats with southern exposures were significantly ahead in terms of egg development compared to eggs collected from microhabitats with eastern exposures (Table 3). However, the question of whether the females of the Altai population truly prefer southern slopes for oviposition remains open. Although in the experiment described here, almost all of the discovered egg masses were concentrated on slopes with a southern exposure, in another area of Altai, one of the authors observed SM eggs located on the northern slope of the rock. However, even on the northern slopes of the rocks, nearby larch trunks (the preferred host tree for the Altai SM population) were free of SM egg masses, which emphasises the preference of this population for rocks rather than trees for egg laying.

Our hypothesis that rock biotopes provide an acceptable temperature for overwintering eggs has also been confirmed. During the entire observation period, the temperature of the rock surface did not fall below −18.5 °C (Table 1), while the minimum air temperature reached a value of −32.2 °C (lethal for SM eggs). The average monthly temperatures of the rock surface were also significantly higher (Table 1). Thus, warmer wintering conditions in rocky microhabitats are critically important for the survival of SMs in low-snow areas of eastern and southern Siberia. In addition, the choice of rocks with a southern exposure accelerates the development of SM caterpillars in spring, which is an additional advantage of rock biotopes.

Small insects (or their eggs) can obtain the greatest benefit from rock biotopes, especially those species that exhibit positive thigmotaxis. This is because close contact with the rock surface is very important for the object to benefit from the warming properties of rock microbiotopes. The importance of close contact of the object with the surface is clearly shown in the results of the simulation experiment. We observed (Figure 6) that even a small air gap (3 cm) between the wall and the object led to a sharp decrease in the warming effect.

## 5. Conclusions

In the course of this study of the preferred wintering microhabitats of the Altai SM population, the following results were obtained. First, it was shown that the average and minimum temperatures of the rock surface, respectively, were 2–5 °C and 4–13 °C higher than the winter air temperature. At the same time, the lower the temperature of the winter air was, the greater the temperature difference between the air and the rock surface. Thus, the microclimate of the rock biotopes remained within the temperature stability range of the wintering SM eggs, which allowed them to survive an extreme (lethal) decrease in air temperature. The preference of rock biotopes by the East Siberian geographical form of the SM was apparently suitable, since it allowed the eggs to survive in regions of Siberia with little snow. Second, it was shown that the close contact of the insects with the rock surface was very important for them to benefit from the warming properties of the rock microhabitats. Apparently, small animals, in particular insect species that exhibit positive thigmotaxis, can receive the greatest benefit from rock biotopes. Third, an independent method confirmed that the lower limit of the temperature stability of the eggs of the Altai population was approximately −30 °C. These results may help reassess the role of the mountain landscape in the spread of insect species.

With increasing international transport activities, the rates of invasive species introductions have also increased. In Canada alone, over 80 invasive alien forest species have been introduced since 1882 [53], which called for research models of their spread. However, the existing forecasts of the SM spread [16,18,23,25,26] do not yet account for the influence of rock biotopes, the presence of which may contribute to the expansion of the SM range to the north. We hope that our data will also be useful for predicting the distribution of other invasive species in regions where there are rock outcrops. The information obtained will also make it possible to refine models of insect outbreak dynamics in areas of their range where there are rock biotopes.

## Figures and Tables

**Table 1:** Monthly temperatures in the main microhabitats in 2019.

Months	Parameters	Sites					
		Air—Weather Station	Air—Near-Rock Layer	Rock—“Recess”	Rock—“Slit”	Rock—“Flat Surface”	Rock ***
October	Mean	2.1	5.3	9.4	9.2	8.4	9.0
	Difference of averages *	−3.2	-	4.1	3.9	3.1	3.7
	Maximum	21.5	29.5	15.2	17.5	37.9	23.5
	Minimum	−20.3	−13.3	0.0	−1.4	−8.8	−3.4
	Fluctuation amplitude **	41.8	42.8	15.2	18.9	46.7	26.9
November	Mean	−8.9	−5.8	−0.8	−1.9	−3.0	−1.9
	Difference of averages *	−3.1	-	5.0	3.9	2.8	3.9
	Maximum	12.5	15.7	11.3	11.2	20.4	14.3
	Minimum	−32.2	−22.5	−9.0	−11.8	−18.5	−13.1
	Fluctuation amplitude **	44.7	38.2	20.3	23.0	38.9	27.4
December	Mean	−10.3	−6.6	−3.3	−4.2	−5.0	−4.2
	Difference of averages *	−3.7	-	3.0	2.4	1.6	2.3
	Maximum	6.6	7.6	0.4	2.3	8.4	3.7
	Minimum	−22.7	−19.0	−8.9	−8.7	−13.7	−10.4
	Fluctuation amplitude **	29.3	26.6	9.3	11.0	22.1	14.1

* Difference of averages—the difference in average temperatures (per month) between the data in the corresponding microhabitats and the data of the “air—near-rock layer” is given. ** Fluctuation amplitude—the difference between maximum and minimum temperatures is given. *** Rock—average values for three rock microhabitats.

**Table 2:** Average minimal temperatures during the winter months (November–December 2019) in the main microbiotopes.

	Sites				
	Air—Near-Rock Layer	Rock—“Recess”	Rock—“Slit”	Rock—“Flat Surface”	Rock *
Average minimal temperature	−10.7	−2.7	−4.1	−6.5	−4.4
Temperature difference	-	8.0	6.6	4.2	6.3
*p*-value **		U = 14, *p* = 6.42 × 10^−7^	U = 32, *p* = 2.342 × 10^−5^	U = 68, *p* = 0.0012	U = 46, *p* = 0.00036

* Rock—average values for three rock microhabitats. ** *p*-value—the results of the analysis according to the criterion U (Mann–Whitney).

**Table 3:** Viability of SM eggs before and after wintering in rock microhabitats.

Parameters	Population before Wintering	After Wintering—Southern Slope			After Wintering—Eastern Slope
		“Flat surface”	“Slit”	“Recess”	“Deep Recess”
Proportion of viable eggs *, %	91 ± 2	84 ± 7	88 ± 3	79 ± 6	82 ± 5
Time before the beginning of hatching, hour	163 ± 7	14 ± 2	27 ± 2	22 ± 1	91 ± 3
*p*-value **	8.72 × 10^−7^	2.48 × 10^−7^	6.76 × 10^−7^	1.26 × 10^−7^	-

* Data are shown as the mean ± standard error of the mean (mean ± s.e.m.). The viability of overwintered eggs in these microhabitats had no significant differences from the viability of eggs before wintering (*t*-test with Bonferroni corrections); data on the level of significance of the differences are not given. ** The probability of coincidence of the timing of the caterpillars beginning to hatch (*t*-test with Bonferroni amendments). The timing of the caterpillars beginning to hatch from the eastern slope differed significantly from that of the specimens wintering on the southern slope.

## Data Availability

See Supplementary Materials (Table S1).

## Supplementary Materials

The following supporting information can be downloaded at: https://www.mdpi.com/article/10.3390/insects13080712/s1, Table S1: Temperature in rock microhabitats.
